# A comprehensive prognostic and immunological implications of Gremlin 1 in lung adenocarcinoma

**DOI:** 10.3389/fimmu.2025.1529195

**Published:** 2025-02-24

**Authors:** Hongyan Li, Yang Zhou, Jiaqing Xiao, Fang Liu

**Affiliations:** ^1^ Department of Medical Oncology, Harbin Medical University Cancer Hospital, Harbin, China; ^2^ Institute of Disinfection and Infection Control, Heilongjiang Provincial Center for Disease Control and Prevention, Harbin, China

**Keywords:** GREM1, lung adenocarcinoma, prognosis, immune analysis, tumor invasion and metastasis

## Abstract

**Background:**

Lung adenocarcinoma (LUAD) is a prevalent form of lung cancer globally, known for its high invasiveness, metastatic potential, and notable heterogeneity, particularly in its response to immunotherapy. Gremlin 1 (GREM1) is implicated in tumor progression and poor prognosis in multiple cancers. However, GREM1’s specific role in LUAD remains unclear. This study systematically examines GREM1 expression in LUAD and its association with tumor progression, immune microenvironment, and prognosis.

**Methods:**

Gene expression data from the TCGA and GSE31210 databases were analyzed using Weighted Gene Co-expression Network Analysis (WGCNA), GO and KEGG enrichment analyses. The prognostic value of GREM1 was evaluated through survival analysis, Cox regression, and Kaplan-Meier curves. Additionally, immune microenvironment analysis was conducted to explore the relationship between GREM1 and immune cell infiltration. *In vitro* experiments, including Western blot and assays for cell proliferation, migration, and invasion, were performed to confirm the specific role of GREM1 in LUAD cells.

**Results:**

GREM1 was significantly upregulated in tumor tissues and correlated with poor prognosis. Moreover, GREM1 was significantly associated with immune cell infiltration and immunotherapy response within the immune microenvironment. *In vitro* experiments confirmed that GREM1 overexpression significantly promoted LUAD cell proliferation, migration, and epithelial-mesenchymal transition (EMT), whereas GREM1 knockdown suppressed these functions.

**Conclusions:**

A comprehensive analysis indicates that GREM1 is crucial in LUAD progression, with its overexpression predicting poor prognosis. GREM1 could be a potential therapeutic target for LUAD, providing insights for personalized therapy optimization.

## 1 Introduction

Lung adenocarcinoma (LUAD) is the most common form of lung cancer worldwide, representing over 40% of all cases (1). The incidence of LUAD has continued to increase in recent years, especially among non-smokers (2). LUAD’s high mortality rate and complex pathogenesis have made it a central focus of lung cancer research and treatment. LUAD progression involves multiple intricate biological mechanisms, such as tumor cell invasion, metastasis, and complex interactions with the host immune system. Although significant advances in molecular targeted therapies and immunotherapies have improved LUAD survival rates, the cancer’s high invasiveness and metastatic nature continue to limit treatment efficacy and patient outcomes (3).

Tumor invasion and metastasis are key features of the malignancy in LUAD. Studies have demonstrated that LUAD cells acquire invasive phenotypes via epithelial-mesenchymal transition (EMT), enabling them to breach the basement membrane and invade the bloodstream or lymphatic system. This process is a crucial step in the dissemination of tumor cells to distant organs (4, 5). Additionally, immune evasion mechanisms within the tumor microenvironment (TME) play a significant role in facilitating LUAD metastasis. Tumor cells can express immune checkpoint molecules, such as PD-L1 which suppress immune system-mediated anti-tumor responses, thereby enhancing their invasiveness and metastatic potential (6, 7).

As our understanding of the biological characteristics of LUAD deepens, immunotherapy strategies have gained significant attention as a treatment option. The introduction of immune checkpoint inhibitors (ICIs) has opened new therapeutic avenues for some LUAD patients, significantly extending their survival (8). However, not all LUAD patients respond to immunotherapy (9). This underscores the need for further investigation into the immune microenvironment of LUAD patients to optimize personalized treatment strategies and improve therapeutic efficacy.

GREM1 (Gremlin 1) is a secreted glycoprotein belonging to the differential screening-selected gene in neuroblastoma (DAN) family, primarily known as an antagonist of bone morphogenetic proteins (BMPs) (10, 11). Previous studies have demonstrated that GREM1 plays a pivotal role in tumor progression, metastasis, and immune regulation across various cancers. In breast cancer, GREM1 is secreted by cancer-associated fibroblasts (CAFs) and promotes an immunosuppressive tumor microenvironment, facilitating tumor invasion and metastasis (12). In pancreatic cancer, GREM1 plays a critical role in maintaining tumor cellular heterogeneity and therapy resistance, leading to increased tumor aggressiveness and poor prognosis (13). Additionally, in colorectal cancer, GREM1 has been implicated in EMT and extracellular matrix (ECM) remodeling, enhancing tumor invasiveness (14). In prostate cancer, GREM1 has been linked to lineage plasticity and therapeutic resistance, allowing tumor cells to evade the effects of targeted therapy (15).

Given its established roles in multiple cancers, it is important to explore the function of GREM1 in lung adenocarcinoma (LUAD). However, its precise role in LUAD remains largely unknown. GREM1 is thought to modulate the TGF-β signaling pathway to induce EMT, enhance invasiveness, and reshape the immune microenvironment (16). Furthermore, recent findings indicate that GREM1 contributes to immune evasion by influencing immune cell infiltration and function (17). These observations suggest that GREM1 may serve as a novel biomarker and potential therapeutic target in LUAD (18).

Despite these insights, the specific function and underlying mechanisms of GREM1 in LUAD remain unclear. Given the high heterogeneity of LUAD and the complex tumor-immune interactions, a comprehensive analysis of GREM1 is warranted to elucidate its prognostic significance and immunoregulatory function. To address this, we systematically investigated GREM1 expression in LUAD, its correlation with prognosis, and its potential involvement in the immune landscape through bioinformatics analysis and experimental validation. Our findings provide novel insights into the biological and clinical implications of GREM1, offering a potential target for personalized therapeutic strategies in LUAD.

## 2 Materials and methods

### 2.1 Data collection

Clinical information and transcriptomic data of LUAD patients were downloaded from The Cancer Genome Atlas (TCGA) database (https://portal.gdc.cancer.gov/), and data from the GES31210 dataset, including transcriptomic, phenotypic, and clinical details, were accessed from the Gene Expression Omnibus (GEO) (NCBI, http://www.ncbi.nlm.nih.gov/geo/). Data were processed in R software (version 4.1.3) using the “limma” package for normalization and log transformation. GREM1 expression levels in normal and tumor tissues were compared using the Wilcoxon test, with statistical significance set at two-tailed *P* < 0.050. To control for potential false positive results arising from testing a large number of genes simultaneously, we applied the Benjamini-Hochberg False Discovery Rate (FDR) correction. This approach ensured the statistical reliability of the identified differentially expressed genes while minimizing the false positive rate to the greatest extent possible.

### 2.2 WGCNA analysis

We conducted a WGCNA on preprocessed gene expression data using the “WGCNA” package in R software (version 4.1.3). Outlier samples were identified and removed as an initial step. Subsequently, we employed the pickSoftThreshold function to test and select an appropriate soft threshold. Gene co-expression modules were identified using the dynamic tree cut method, and the module eigengenes (MEs) for each module were calculated. Finally, Pearson correlation analysis and Student’s *t*-tests were conducted to evaluate relationships between module eigengenes and patient clinical characteristics.

### 2.3 Functional enrichment analysis

Gene annotation was performed using the “org.Hs.eg.db” package in R, with the additional installation of “ggplot2”, “colorspace”, “stringi”, “clusterProfiler”, and “enrichplot” packages. The “enrichGO” function from the “clusterProfiler” package was used for GO enrichment analysis, while the “enrichKEGG” function was used for KEGG enrichment analysis. In the GO enrichment analysis and KEGG pathway functional enrichment analysis, we tested the significance of multiple biological pathways and functions. To address statistical errors caused by multiple hypothesis testing, we also applied FDR correction to adjust the p-values. This method ensured the statistical robustness of the enrichment analysis results. The results of the enrichment analyses were visualized using circular plots.

### 2.4 Survival and prognostic analysis

Patients were classified into high- and low-risk groups according to the median expression level of the GREM1 gene. The association between GREM1 expression and OS was illustrated with Kaplan-Meier survival curves, and the log-rank test was applied to calculate survival differences between the two groups. Univariate and multivariate Cox regression analyses were performed using the “survival” and “forestplot” R packages to calculate hazard ratios (HR) and 95% confidence interval (CI), enabling assessment of GREM1’s prognostic value. The Cox regression model included GREM1 expression levels and other clinical covariates potentially influencing survival (e.g., age, gender, and tumor stage) to assess its independent prognostic value through multivariate analysis. Since survival analysis primarily focused on single hypothesis testing, multiple comparison corrections were not applied.

### 2.5 Construction and evaluation of the nomogram

Based on the expression levels of GREM1 and clinical stage, a nomogram was constructed using the “rms” R package to predict the OS probabilities for patients with LUAD at 1, 3, and 5 years. This method provides an effective and convenient approach for estimating individual survival rates in patients. Calibration curves were also plotted to assess the nomogram’s predictive accuracy.

### 2.6 Correlation analysis of GREM1 expression with the TME

The “estimate” R package and the ESTIMATE algorithm were used to analyze differences in stromal and immune scores across samples. Bioinformatics tools, including TIMER 2.0 and CIBERSORT, assessed the levels of 22 infiltrating immune cell subtypes. The “limma” and “CIBERSORT” R packages were used to quantify specific cell types in the samples based on overall expression data. In exploring the relationship between GREM1 gene expression and immune cell infiltration (such as T cells and macrophages) as well as immune scores (e.g., ESTIMATE scores), Spearman correlation coefficients were calculated. To minimize the impact of multiple comparisons on the results, *P*-values from the correlation analysis were adjusted using FDR correction.

### 2.7 Correlation analysis of GREM1 with TMB and MSI

We used the “maftools” package in R to process single-nucleotide variant (SNV) data from the TCGA database and calculated each sample’s TMB and MSI. Spearman correlation analysis was conducted to determine the association between GREM1, TMB, and MSI. Additionally, the “fmsb” package was used to create radar charts to visualize the correlation analysis results for TMB and MSI.

### 2.8 Patients and samples

Fresh tissue samples were collected from five LUAD patients who underwent surgical treatment at Harbin Medical University Cancer Hospital. None of the patients received anticancer therapy prior to surgery. This study was approved by the Ethics Committee Review Board of the Harbin Medical University Cancer Hospital and in conformity to the Declaration of Helsinki. Informed written consent was obtained from all patients before participation in the study.

### 2.9 Cell culture

Human LUAD cell lines (PC-9, A549, H1299, H1650, HCC827) and HBE cells were obtained from the Laboratory of Medical Genetics (Department of Biology, Harbin Medical University, Harbin, China). The HBE, A549, H1299, H1650, and HCC827 cell lines were cultured in RPMI 1640 (Gibco, Life Technologies, California, USA), while the PC-9 cell line was cultured in DMEM (Gibco, Life Technologies, California, USA). Both culture media were supplemented with 10% fetal bovine serum (ScienCell, California, USA) and 1% penicillin-streptomycin (PYG0016, Boster). Cell culture was performed in a humidified incubator at 37°C with 5% CO2.

### 2.10 Western blot analysis

Total protein was extracted from LUAD specimens and cells using RIPA buffer (Beyotime, China). Equal amounts of protein were subjected to electrophoresis on 10% SDS-PAGE and transferred to a PVDF membrane. The membrane was blocked with 5% non-fat dry milk (Beyotime, China) in TBST for 1 hour at room temperature, followed by overnight incubation at 4°C with primary antibodies against GREM1 (1:1000, Affinity), E-cadherin (1:20000, Proteintech), N-cadherin (1:3000, Proteintech), Vimentin (1:50000, Proteintech), and GAPDH (1:1000, Absin). After washing with TBST, the membrane was incubated with secondary antibodies (1:10000, Beijing Zhongshan Golden Bridge Biotechnology Co., Ltd.) for 1 hour at room temperature. Finally, target protein bands were detected using the ECL detection system (Beyotime, China), with GAPDH serving as the internal controls.

### 2.11 Proliferation, migration, and invasion assays

A549 cells were transfected with GREM1-OE (RiboBio, China) according to the manufacturer’s instructions to upregulate GREM1 expression. H1650 cells were transfected with GREM1 siRNA (RiboBio, China) as per the manufacturer’s guidelines to downregulate GREM1 expression.

The proliferation of A549 and H1650 cells was assessed using the Cell Counting Kit-8 (CCK-8) (APExBIO, USA). Approximately 4 × 10³ cells were seeded into 96-well plates and cultured for 24, 48, 72, and 96 hours. Optical density (OD) at 450 nm was measured with a microplate reader.

In the wound healing assay, when the cells reached confluence, we created wounds in the cell layer using a 1000 µL pipette tip. Serum-free medium was then applied to maintain cell viability. Wound areas were photographed immediately after wounding (0 hours) and 24 hours later using an optical microscope (Nikon, Japan).

In the Transwell assay, 2 × 10^4^ cells were seeded into each upper chamber of 24-well plates, with or without Matrigel (Becton Dickinson). After 24 or 48 hours of incubation (with or without Matrigel), cells on the bottom membrane were fixed with paraformaldehyde, stained with crystal violet, and analyzed microscopically.

### 2.12 Statistical analysis

Statistical analyses were conducted using R version 4.1.3. Survival curves were generated with the Kaplan-Meier method and analyzed statistically. The Wilcoxon test was used to compare differences between groups, while Pearson and Spearman correlation coefficients were calculated for association analyses. To control for multiple comparisons, the Benjamini-Hochberg False Discovery Rate (FDR) correction was applied when analyzing differentially expressed genes and pathway enrichment analyses. Statistical significance was defined as a two-tailed *P* value of < 0.050.

## 3 Results

### 3.1 Construct a gene co-expression network and select key genes

**Figure 1:**
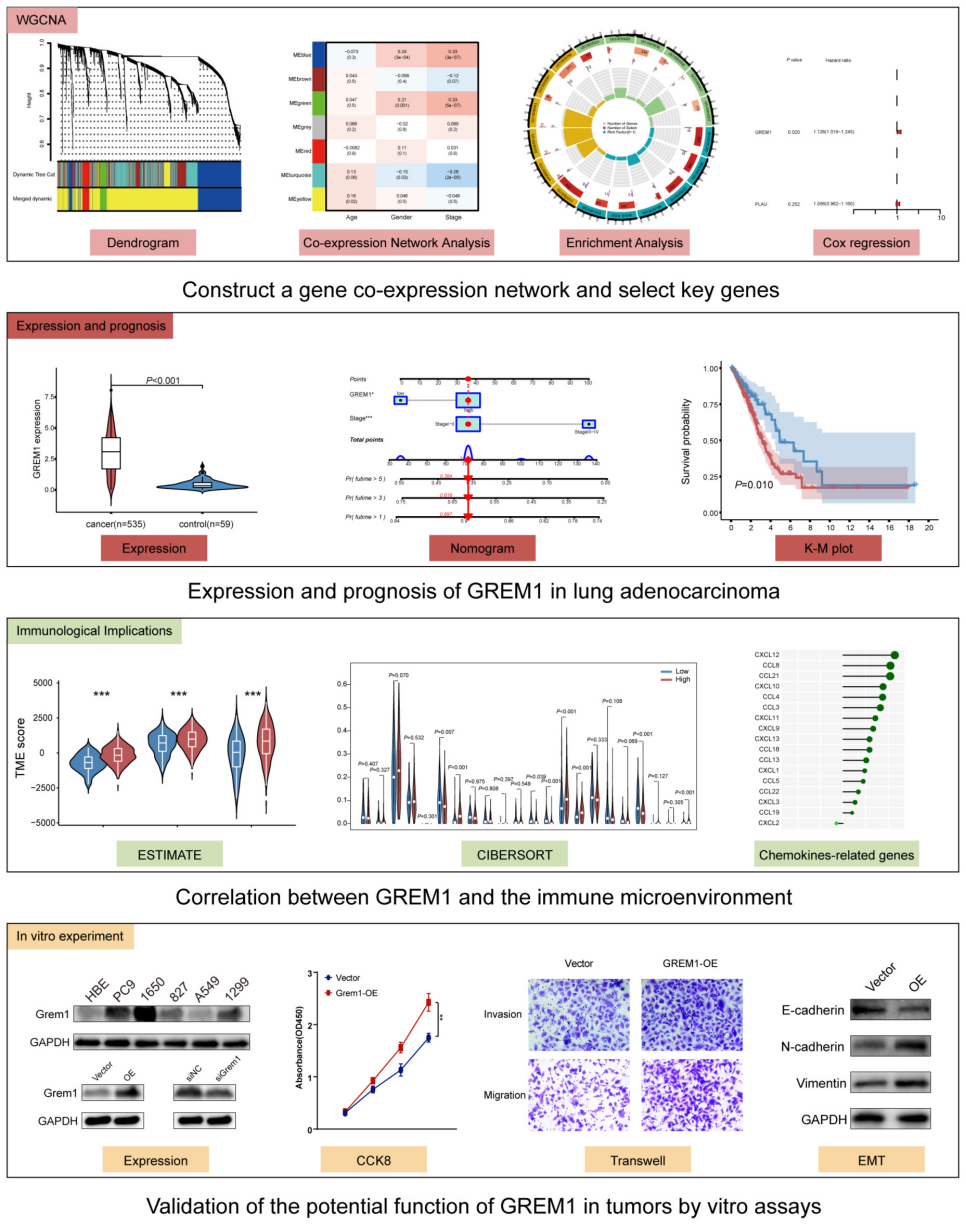
Overview of the study design for the identification of GREM1, a key gene associated with the invasiveness and metastatic potential of LUAD and promotes tumor immune evasion. (***P < 0.001).

First, we summarized the research design workflow, including the screening process of GREM1 and the exploration of its biological function (**Figure 1**).

**Figure 2:**
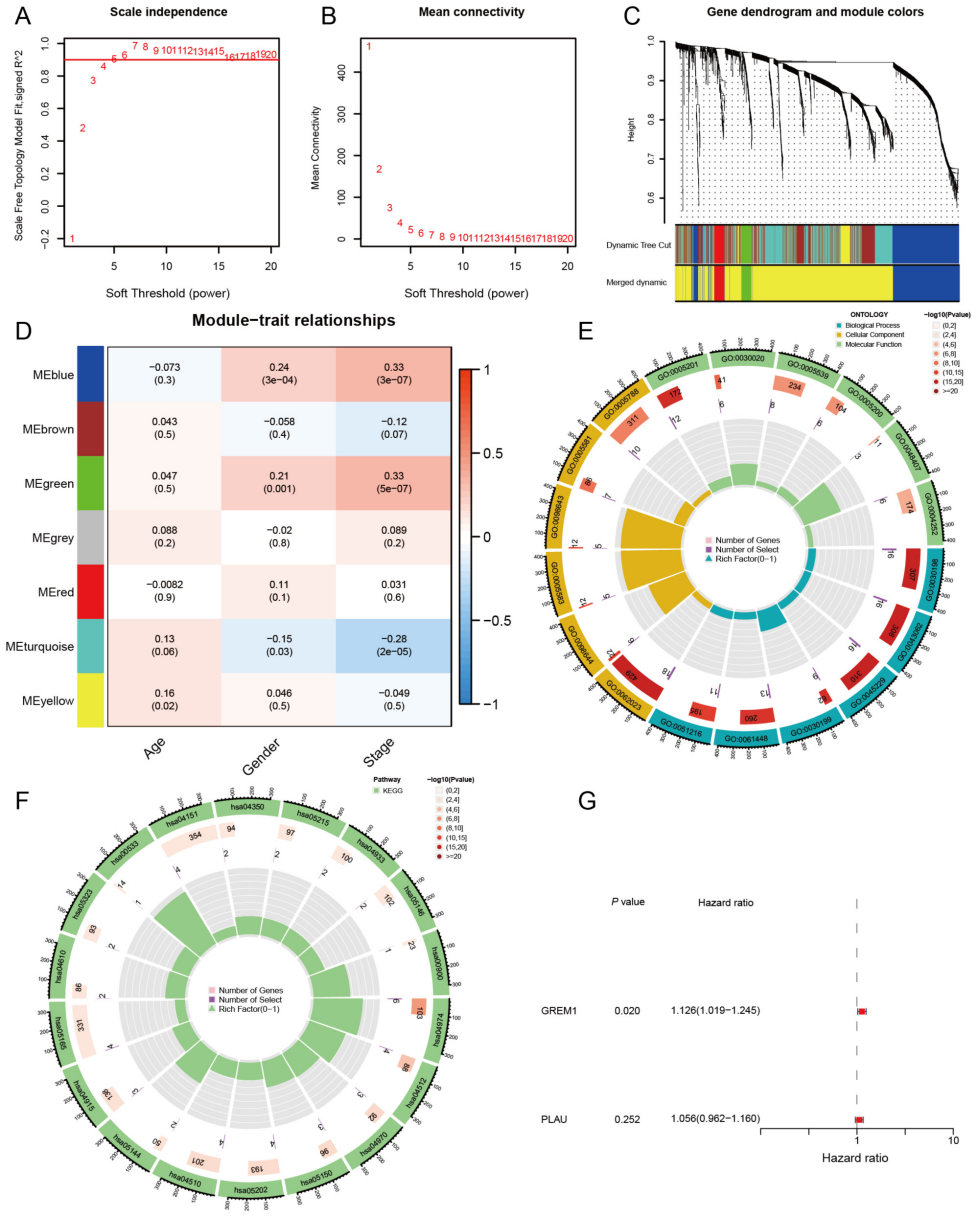
Construction of key gene modules and annotation of biological functions and pathways. **(A, B)** Analysis of the scale-free fit index and the mean connectivity for various soft thresholding powers (β). **(C)** A clustering dendrogram formed by weighted correlation coefficients, clustering genes with similar expression patterns into co-expression modules, with each color representing a module. **(D)** Heatmap of the correlation between module eigengenes (MEs) and age, gender, as well as disease stage. **(E, F)** The top biological pathways of the green module genes. **(G)** Univariate Cox analysis of OS for 2 genes in LUAD from TCGA.

Differential gene expression analysis was performed between LUAD tissues and normal tissues using the TCGA and GSE31210 datasets. In the TCGA dataset, 4,859 differentially expressed genes were identified (|log2(FC)| > 1, adj.P.Val < 0.05), of which 2,347 were upregulated and 2,512 were downregulated. Similarly, in the GSE31210 dataset, 3,315 differentially expressed genes were detected (|log2(FC)| > 1, adj.P.Val < 0.05), with 1,785 upregulated and 1,530 downregulated. To identify key shared differentially expressed genes across both datasets, we conducted an intersection analysis, revealing 1,927 common genes. We then integrated clinical data, including age, sex, and disease stage, and performed weighted gene co-expression network analysis (WGCNA) on 1,927 differentially expressed genes. The scale-free fit index and average connectivity were calculated to determine a soft threshold of β=4 (**Figures 2A, B**). Using the dynamic tree cut method, we identified seven gene co-expression modules, as shown in the clustering tree in **Figure 2C**. The module-trait correlation heatmap (**Figure 2D**) revealed a significant correlation between the green module and the clinical stage of LUAD (cor=0.33, *P*=5e-07), suggesting that the 72 genes in this module may be closely associated with LUAD invasion and metastasis. Consequently, the green module was identified as the key module for further investigation.

To further explore the biological functions of genes in the green module, we conducted Gene Ontology (GO) analysis (**Figure 2E**). The results of the biological process (BP) analysis revealed that these genes are strongly associated with the organization of the extracellular matrix and extracellular structures. In terms of cellular components (CC), these genes participate in the formation of collagen-containing extracellular matrices and the lumen of the endoplasmic reticulum. Regarding molecular functions (MF), these genes contribute to activities such as the structural composition of extracellular matrix components. Through KEGG enrichment analysis (**Figure 2F**), we observed that genes within the green module are significantly enriched in essential biological pathways, such as protein digestion and absorption, ECM-receptor interaction, and the PI3K-Akt signaling pathway. These findings indicate that genes in the green module may impact tumor cell invasion and metastasis by modulating the formation and function of the TME, thus playing a crucial role in the progression and pathology of LUAD.

In this study, we selected the top 25 genes with the highest connectivity from the 72 genes in the green module and performed an intersection analysis with 2,660 genes from the immune database. This analysis identified two candidate genes: GREM1 and PLAU. To evaluate the prognostic impact of these two genes on LUAD patients, we conducted univariate Cox regression analysis (**Figure 2G**). The results showed that, of the two candidate genes, only the expression level of GREM1 was significantly associated with LUAD patient prognosis. (*P* = 0.020, HR = 1.126, 95% CI: 1.019–1.245).

### 3.2 Expression and prognosis of GREM1 in LUAD

**Figure 3:**
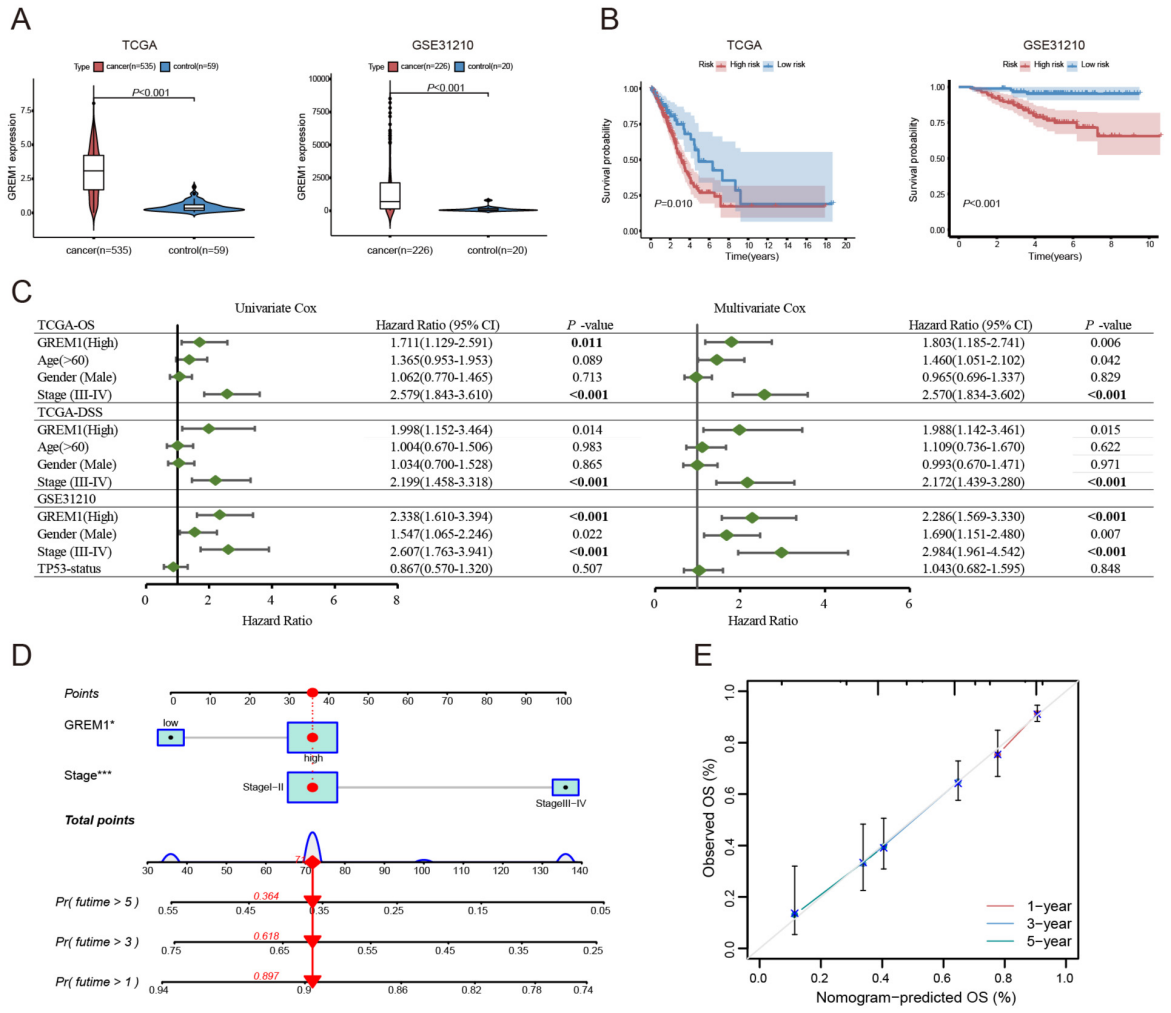
The expression levels and prognostic significance of GREM1 in LUAD. **(A)** Boxplots showing significantly higher GREM1 expression in tumor tissues compared to non-tumor tissues in the TCGA and GSE31210 datasets (*P* < 0.001). **(B)** Kaplan-Meier survival curves demonstrating that LUAD patients with high GREM1 expression have significantly shorter OS in both datasets. **(C)** Forest plot summarizing the results of univariate and multivariate Cox regression analyses for OS and disease-specific survival (DSS) in TCGA and GSE31210, with GREM1 as an independent prognostic factor. **(D)** Nomogram integrating GREM1 expression and clinical stage to predict 1-, 3-, and 5-year OS for LUAD patients, based on the TCGA-LUAD dataset. **(E)** Calibration curves illustrating the predictive accuracy of the nomogram for 1-, 3-, and 5-year OS in the TCGA-LUAD dataset, closely aligned with ideal predictions. (*P < 0.05, ***P < 0.001).

We assessed the prognostic impact of GREM1 in LUAD patients across multiple datasets. In the TCGA-LUAD dataset, which includes 594 patient samples (535 tumor samples and 59 normal samples), GREM1 expression levels were significantly higher in tumor tissues compared to normal tissues (*P*<0.001). Similarly, in the LUAD dataset GSE31210, which comprised 246 patient samples (226 tumor samples and 20 normal samples), GREM1 expression was significantly higher in tumor tissues (*P*<0.001) (**Figure 3A**). Additionally, we conducted Kaplan-Meier survival analysis to compare overall survival (OS) between patients with high and low GREM1 expression in the TCGA and GSE31210 datasets. The analysis revealed that patients with high GREM1 expression had significantly shorter OS (*P*<0.05) (**Figure 3B**).

To further assess the prognostic and diagnostic significance of GREM1 in LUAD, we conducted univariate and multivariate Cox regression analyses. Univariate Cox analysis revealed a significant association between high GREM1 expression and prognosis. In the multivariate Cox proportional hazards regression model for the TCGA and GSE31210 datasets, high GREM1 expression was confirmed as an independent prognostic biomarker (for OS, HR=1.803 and 2.286, 95% CI=1.185-2.741 and 1.569-3.330, *P*=0.006 and <0.001, respectively; for disease-specific survival (DSS), HR=1.988, 95% CI=1.142-3.461, *P*=0.015) (**Figure 3C**).

To improve prediction of the clinical prognostic value of GREM1, we incorporated GREM1 expression and tumor staging-both independent mortality predictors-into a multivariable Cox proportional hazards model. Using this model, we developed a prognostic nomogram to predict the OS of LUAD patients at 1, 3, and 5 years (**Figure 3D**), achieving predictive accuracies of 0.897, 0.618, and 0.364, respectively. This model visually represents the combined impact of GREM1 expression and tumor staging on LUAD prognosis. We evaluated and validated this nomogram using TCGA data. Calibration curve analysis confirmed that the model provides reliable accuracy in predicting 1-, 3-, and 5-year OS, aligning with the standards of an ideal predictive model (**Figure 3E**).

### 3.3 Correlation between GREM1 and the immune microenvironment

**Figure 4:**
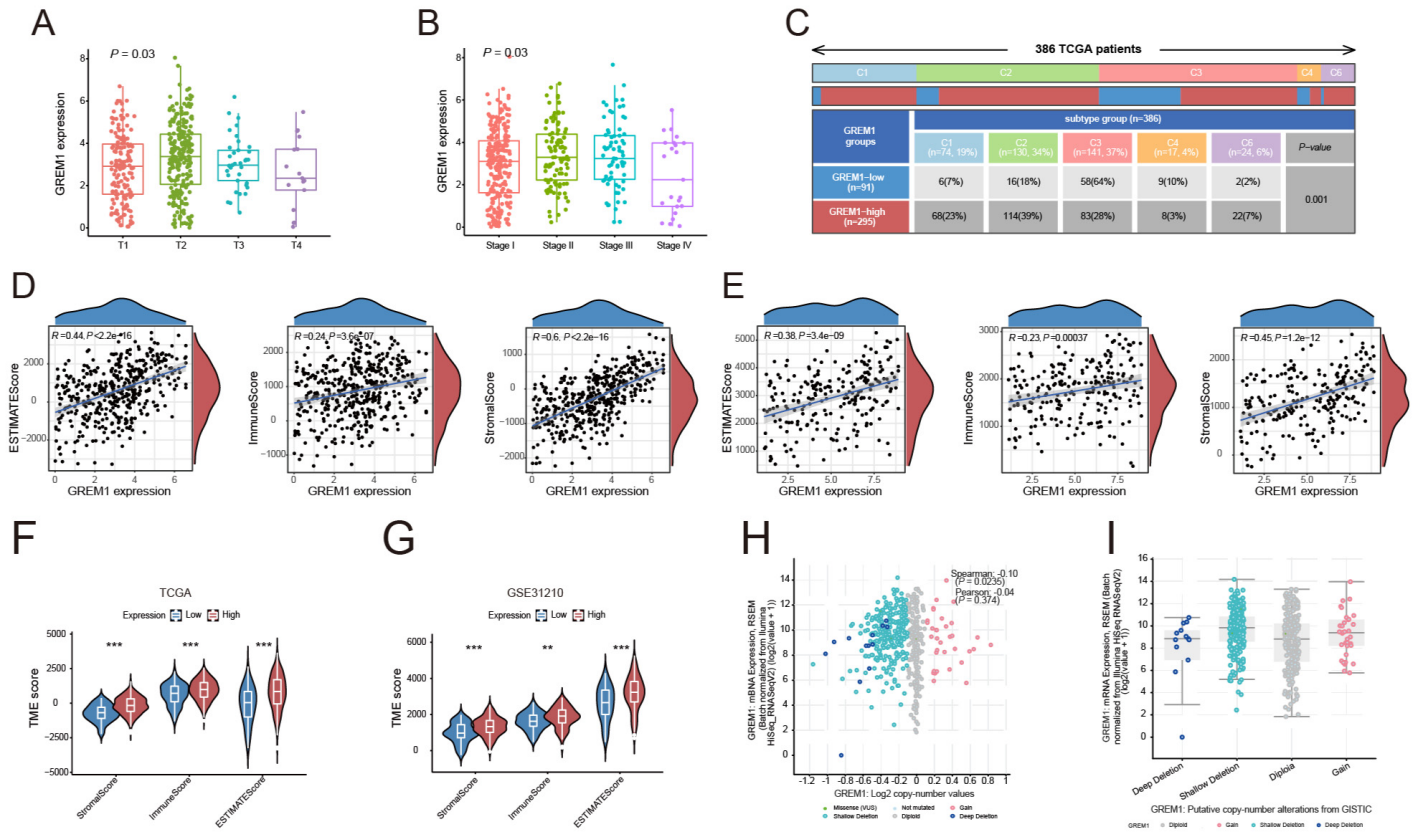
The relationship between GREM1 and the tumor immune microenvironment. **(A, B)** Boxplots illustrating the association between GREM1 expression and tumor status and clinical stages in the TCGA-LUAD dataset. GREM1 expression increases significantly with tumor progression. **(C)** Heatmap showing the distribution of GREM1 expression across six immune subtypes. **(D, E)** Scatter plots showing the positive correlation of GREM1 expression with the ESTIMATE score, Immune score, and Stromal score in TCGA-LUAD and GSE31210 datasets. **(F, G)** Violin plots comparing the ESTIMATE score, Immune score, and Stromal score between high- and low-GREM1 expression groups in TCGA-LUAD and GSE31210, with significant differences observed. **(H)** Scatter plot demonstrating a positive correlation between GREM1 mRNA expression levels and Log2 copy number variations in TCGA-LUAD, suggesting genomic alterations contribute to elevated expression. **(I)** Boxplot showing GREM1 expression levels across different copy number alteration categories from the GISTIC dataset in TCGA-LUAD, further emphasizing its association with genomic instability. (**P < 0.01, ***P < 0.001).

Initially, we evaluated the role of GREM1 in the invasion and metastasis of LUAD. We categorized the samples using the MEXPRESS tool based on several clinical factors, including the occurrence of new tumors post-treatment, tumor stage, smoking history, and sample type. The results revealed significant differential expression of GREM1 related to tumor status (*P*=0.03) and staging (*P*=0.03), with significantly higher expression levels in stages T2 and T3 (**Figure 4A**). Furthermore, GREM1 expression was significantly higher in stage II and III patients than in stage I patients (**Figure 4B**). This finding is consistent with our previous studies, which suggest that GREM1 expression is closely linked to LUAD tumor progression. Although the GREM1 expression differences in T4 and stage IV did not reach statistical significance, this may be attributed to an insufficient sample size.

We next analyzed GREM1 expression across six immune subtypes to investigate its relationship with the intratumoral immune state (**Figure 4C**). The results revealed a significant upregulation of GREM1 expression in the wound healing, IFN-gamma dominant, and immune-related inflammation subtypes. These findings suggest that GREM1 is not only involved in the invasion and metastasis of LUAD but also closely associated with the TME and immune response.

To further explore the impact of GREM1 expression on the TME, we compared its correlation with the ESTIMATE score, Immune score, and Stromal score. GREM1 expression in the TCGA and GSE31210 datasets was significantly positively correlated with the immune score (*P*=3.6e-07, R=0.24; *P*=0.00037, R=0.23), stromal score (*P*<2.2e-16, R=0.6; *P*=1.2e-12, R=0.45), and estimate score (*P*<2.2e-16, R=0.44; *P*=3.4e-09, R=0.38) (**Figures 4D, E**). Additionally, significant differences in these scores were observed between the high and low GREM1 expression groups (**Figures 4F, G**). These results suggest that GREM1 overexpression may promote tumor growth and metastasis by modifying the physical or chemical properties of the TME.

A significant positive correlation was observed between GREM1 mRNA expression levels and Log2 copy number variation, suggesting that increased copy numbers are associated with elevated GREM1 expression (*P*=0.0235, R=0.10) (**Figure 4H**). Further analysis of GREM1 gene alterations in TCGA-LUAD tissues, including deep and shallow deletions, diploidy, and copy number gains, indicated a marked increase in GREM1 copy number (**Figure 4I**).

**Figure 5:**
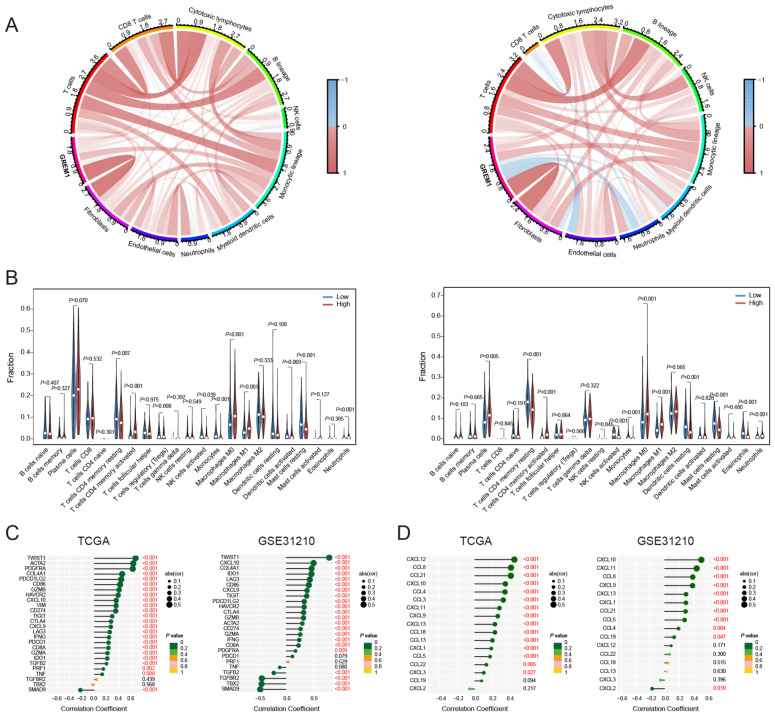
Correlation of GREM1 expression with immune cells and chemokines in LUAD. **(A)** Chord diagrams showing the correlation of GREM1 expression with various immune cell types in the TCGA cohort (left) and the GSE31210 cohort (right). The color intensity represents the strength of the correlation, with red indicating positive correlations and blue indicating negative correlations. **(B)** Bar plots displaying the differences in immune cell fractions between GREM1 high and low expression groups in the TCGA cohort (left) and the GSE31210 cohort (right). **(C)** Correlation analysis between GREM1 expression and immune-related genes in the TCGA (left) and GSE31210 (right) cohorts. **(D)** Correlation analysis of GREM1 expression with chemokine gene signatures in the TCGA (left) and GSE31210 (right) cohorts.

Based on the above results, we applied the MCP counter algorithm to examine the association between GREM1 and immune cells. Comparative analysis of the TCGA and GSE31210 datasets demonstrated a significant association between GREM1 expression and the infiltration of multiple immune cells (**Figure 5A**). Following this, the relationship between GREM1 expression and 22 immune cell types was analyzed using the CIBERSORT algorithm (**Figure 5B**). Notably, significant differences in the infiltration levels of specific immune cells, including CD4 memory resting T cells, CD4 memory activated T cells, activated NK cells, monocytes, macrophages, resting mast cells, and neutrophils, were observed between high and low GREM1 expression groups. These findings indicate that GREM1 may affect immune cell composition within the tumor immune microenvironment (TIME).

To explore the potential gene networks or pathways associated with GREM1, we examined its correlations with other genes. As shown in **Figure 5C**, in the TCGA dataset, GREM1 exhibited a significant positive correlation with twist family BHLH transcription factor 1 (TWIST1), actin alpha cardiac muscle 2 (ACTA2), and platelet-derived growth factor receptor alpha (PDGFRA) (R>0.6, *P*<0.001), and a significant negative correlation with SMAD family member 9 (SMAD9) (R<-0.2, *P*<0.001). In the GSE31210 dataset, GREM1 showed a significant positive correlation with TWIST1 (R>0.6, *P*<0.001), and a significant negative correlation with transforming growth factor beta receptor 2 (TGFBR2), T-Box transcription factor 2 (TBX2), and SMAD9 (R<-0.5, *P*<0.001). The chemokine correlation analysis (**Figure 5D**) showed that GREM1 had a strong positive correlation with CXCL12, CCL8, and CCL21 in the TCGA dataset (R>0.4, *P*<0.001). Similarly, in the GSE31210 dataset, GREM1 was positively correlated with CXCL10 and CXCL11 (R>0.4, *P*<0.001).

**Figure 6:**
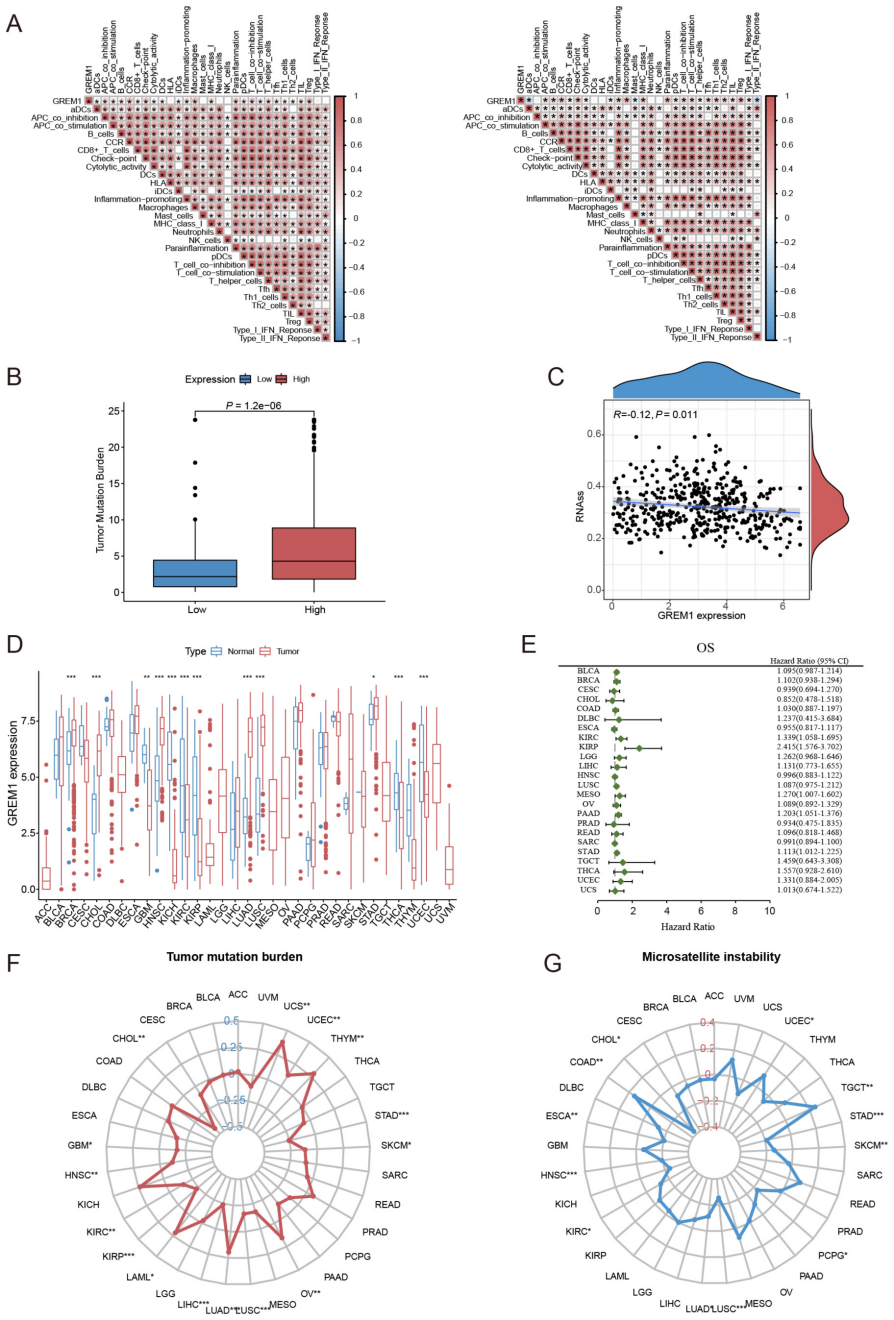
The correlation between GREM1 expression, immune function, and key tumor characteristics across multiple cancers. **(A)** The correlation analysis between GREM1 expression and immune function scores in the TCGA cohort (left) and the GSE31210 cohort (right). **(B)** The difference in TMB between the high and low GREM1 expression groups. **(C)** The correlation analysis between GREM1 expression and RNAss. **(D)** The pan-cancer analysis of GREM1 expression. **(E)** The prognostic analysis of GREM1 in 24 solid tumors. **(F, G)** The analysis of TMB and MSI of GREM1 in pan-cancer. (*P < 0.05, **P < 0.01, ***P < 0.001.).

Furthermore, we examined the effect of GREM1 on clinical responses to ICIs in LUAD. Correlation analysis revealed that higher GREM1 expression was strongly associated with elevated immune function scores, such as C-C chemokine receptor (CCR), checkpoint markers, inflammation-promoting factors, macrophages, T-cell co-inhibition, tumor-infiltrating lymphocytes (TILs), and regulatory T cells (Tregs) (**Figure 6A**). Furthermore, patients with high GREM1 expression exhibited a significantly higher tumor mutation burden (TMB) than those with low expression (*P*=1.2e-06) (**Figure 6B**). Additionally, as GREM1 expression increased, the RNA stemness score (RNAss) gradually decreased (*P*=0.011, R=-0.12) (**Figure 6C**). In conclusion, GREM1 may play a critical role in immune cell recruitment and the response to immunotherapy.

Considering the current scarcity of research on GREM1 in cancer studies, particularly regarding solid tumors, we conducted a systematic evaluation of GREM1 expression across various solid tumor types. Results indicate that GREM1 is highly expressed in LUAD and is overexpressed in other cancers such as BRCA, HNSC, KIRC, KIRP, THCA, and UCEC (**Figure 6D**). Univariate Cox analysis further confirmed the prognostic significance of GREM1 in multiple solid tumors beyond LUAD, such as KIRC, KIRP, MESO, PAAD, and STAD (**Figure 6E**). Analysis of GREM1’s TMB and microsatellite instability (MSI) revealed a strong association between its expression and both the proliferative and metastatic potential of LUAD, along with its response to immunotherapy (**Figures 6F, G**).

### 3.4 Validation of the potential function of GREM1 in tumors by vitro assays

**Figure 7:**
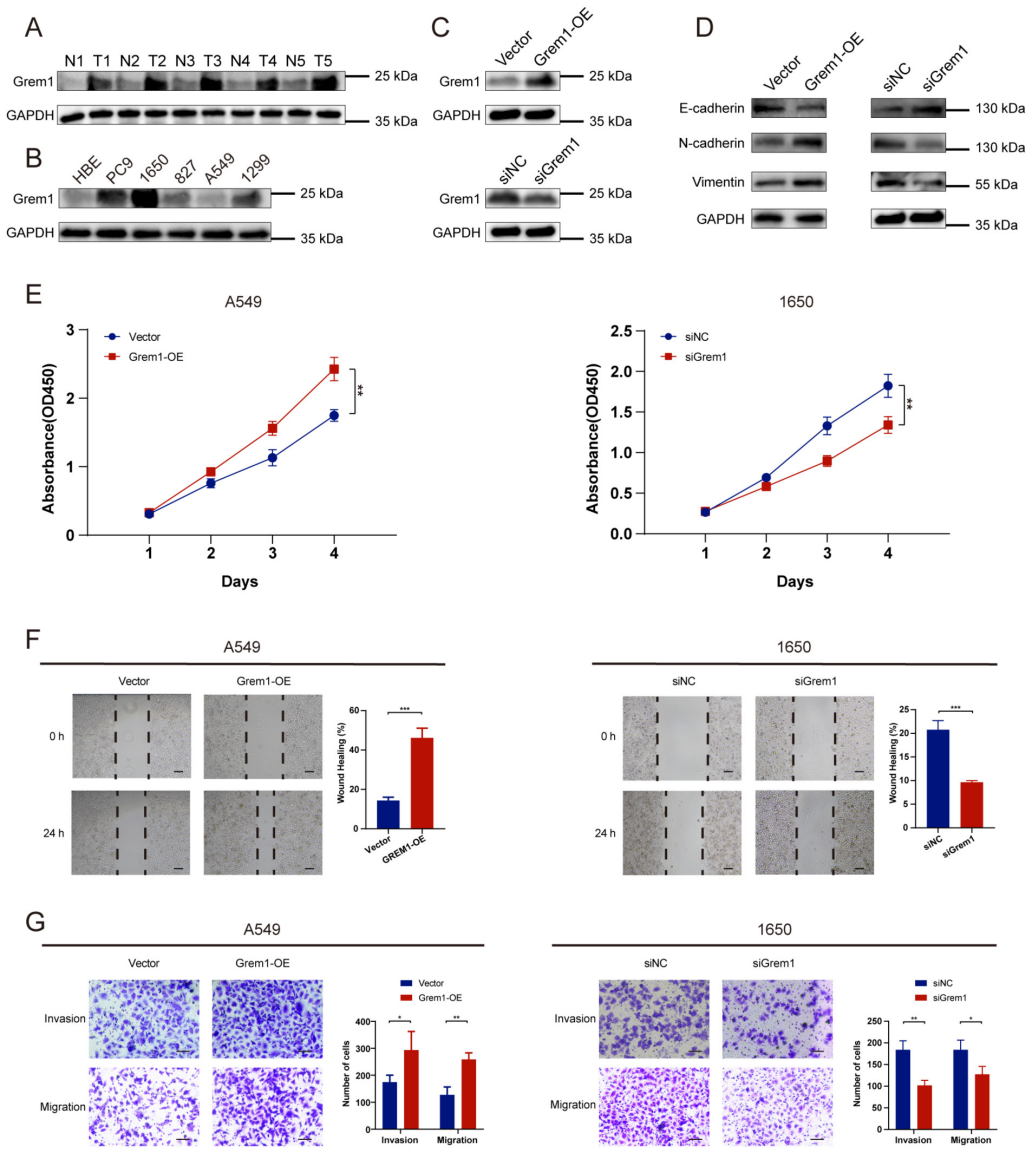
Validation of GREM1’s potential function in LUAD through *in vitro* assays. **(A)** GREM1 expression in five paired tumor tissues (T) and their adjacent normal tissues (N). **(B)** Comparison of GREM1 expressions in HBE and different LUAD cell lines. **(C)** Western blot analysis of GREM1 overexpression and knockdown at protein level in A549 and H1650 cells. **(D)** Western blot analysis of the effects of GREM1 overexpression and knockdown on EMT-related proteins in A549 (left) and H1650 (right) cells respectively. **(E–G)** Overexpression and knockdown of GREM1 inhibited the proliferation, migration and invasion of lung cancer cells by CCK-8 assay **(E)**, wound-healing assay **(F)** and Transwell assay **(G)**. (*P < 0.05, **P < 0.01, ***P < 0.001).

We conducted *in vitro* experiments to investigate the pathogenic mechanism of GREM1 in LUAD. First, we assessed GREM1 expression in tissue samples using Western blot analysis. The results showed significantly higher GREM1 expression in cancerous tissues compared to adjacent non-cancerous tissues in five paired samples (**Figure 7A**). We then collected various cell lines, including LUAD cell lines (PC-9, A549, H1299, H1650, HCC827) and a human bronchial epithelial cell line (HBE), to analyze GREM1 expression levels using Western blot. The results demonstrated generally higher GREM1 expression in LUAD cell lines compared to normal cells (**Figure 7B**). Based on these findings, we selected the H1650 cell line, which showed the highest GREM1 expression, and the A549 cell line, which showed the lowest expression, for further experiments.

A549 cells were transfected with the GREM1 overexpression plasmid. Western blot analysis confirmed that GREM1 levels were significantly higher in the GREM1 overexpression group compared to the control group. A GREM1-targeting siRNA was designed and transfected into H1650 cells, while non-targeting siRNA was used as a control. Western blot results showed that GREM1 expression in the knockdown group was significantly lower than in the control group (**Figure 7C**).

**Figure 7D** shows the effect of GREM1 on EMT-related proteins in A549 and H1650 cells as observed through Western blot analysis. The effects of GREM1 overexpression or silencing on the expression of the epithelial marker E-cadherin and mesenchymal markers N-cadherin and Vimentin were examined. In A549 cells, overexpression of GREM1 significantly downregulated E-cadherin while upregulating N-cadherin and Vimentin, suggesting that GREM1 overexpression promotes the EMT process. Conversely, in H1650 cells, GREM1 knockdown significantly upregulated E-cadherin and downregulated N-cadherin and Vimentin, indicating that GREM1 inhibition may hinder EMT. These findings highlight GREM1’s regulatory role in EMT: its overexpression promotes EMT, while its knockdown suppresses the process. This suggests that GREM1 may influence tumor cell migration and invasion through EMT modulation.

CCK-8 assays were conducted on A549 and H1650 cell lines to evaluate the effect of GREM1 on cell proliferation. The results showed that GREM1 overexpression significantly promoted cell proliferation in A549 cells compared to the control group. Conversely, GREM1 knockdown in H1650 cells significantly decreased proliferation (*P*<0.01) (**Figure 7E**), suggesting that GREM1 is crucial for tumor cell proliferation.

Wound healing assay results indicate that GREM1 overexpression significantly promotes cell migration in A549 cells. At 24 hours, the wound closure rate in the GREM1 overexpression group was significantly higher than in the control group, suggesting that GREM1 enhances A549 cell migration. Similarly, in H1650 cells, GREM1 knockdown significantly reduced cell migration, with the wound closure rate in the siGREM1 group lower than in the control group at 24 hours, indicating that GREM1 downregulation suppresses H1650 cell migration (**Figure 7F**).

Transwell assay results demonstrated that GREM1 expression in A549 and H1650 cells is closely associated with invasion and migration. In A549 cells, GREM1 overexpression significantly enhanced invasive and migratory abilities, as evidenced by more cells passing through the chamber in the overexpression group than in the control group. Conversely, in H1650 cells, GREM1 knockdown significantly inhibited invasion and migration, with fewer cells passing through the chamber in the siGREM1 group than in the control group (**Figure 7G**). These findings suggest that GREM1 positively regulates cell invasion and migration.

## 4 Discussion

In the treatment of LUAD, tumor invasion and metastasis often lead to poor patient prognosis (19). For LUAD patients lacking genetic mutations or those with acquired resistance to targeted therapies, ICIs have become one of the standard treatment options (20). However, the high heterogeneity of tumors poses major challenges to the effectiveness of immunotherapy (21). To gain deeper insights into the progression of LUAD and its response mechanisms to immunotherapy, we applied WGCNA to identify key modules linked to tumor invasion and metastasis, followed by core gene screening, survival analysis, immune cell infiltration analysis, and *in vitro* validation. Our results suggest that GREM1 may serve as a valuable biomarker for survival prognosis and as an indicator for immunotherapy in LUAD.

Analysis of independent LUAD cohorts revealed significantly shorter OS in patients with high GREM1 expression. This observation aligns with findings from studies on GREM1 in other tumor types, such as BRCA, PAAD, and PCa (12, 13, 22, 23). Additionally, a nomogram integrating GREM1 expression and tumor staging showed strong prognostic predictive ability. To further clarify GREM1’s role in cancer prognosis, we performed a pan-cancer analysis in the TCGA cohort, showing that high GREM1 expression is strongly associated with poor prognosis across multiple cancer types, especially in tumors with high TMB and substantial immune cell infiltration (24, 25).

The imbalance in the tumor microenvironment (TME) may contribute to the poor prognosis linked to GREM1 in cancer, as TME status directly affects immune cell infiltration (26). Our research shows a strong positive correlation between GREM1 and immune cell infiltration within tumors. Additionally, GREM1 is enriched across multiple tumor subtypes, including wound healing, IFN-gamma dominant, and immune-related inflammation subtypes, and is closely associated with the abundance of CCR, checkpoint molecules, inflammation-promoting factors, macrophages, T-cell co-inhibition, TIL, and Tregs. This suggests that GREM1 is intricately linked to the remodeling processes within the TME. Previous studies indicate that GREM1 modulates TME by inhibiting BMP signaling, altering cancer cell growth, differentiation, and stromal-immune interactions, thereby driving tumor progression and metastasis (27, 28). Further studies indicate that GREM1, as a key regulatory factor, enhances the supportive role of tumor-associated mesenchymal stem cells (T-MSCs) in tumor cell invasion and metastasis (29).

Furthermore, an analysis of classical immunotherapy response indicators, including TMB and MSI, revealed that patients with elevated GREM1 expression, increased TMB, and higher levels of immune cell infiltration are more likely to benefit from immunotherapy. GREM1 may also impact immunotherapy efficacy by influencing tumor genetic stability (30, 31). Although these findings require further investigation for validation, GREM1 is undoubtedly closely related to the immune microenvironment and immunotherapy response in LUAD.

The association of GREM1 with antitumor immunity may involve multiple complex mechanisms. Previous studies indicate that GREM1 is strongly associated with immune cell infiltration, including M2 macrophages and Tregs, which may promote immune evasion (32). Additionally, GREM1 contributes to T cell exhaustion by upregulating immune checkpoints like CTLA-4 and PD-1 and overactivating key signaling pathways, thereby impairing antitumor immunity and accelerating tumor progression (33, 34). Our study suggests that GREM1 is closely linked to EMT and the inhibition of the TGF-β signaling pathway (14, 35, 36). GREM1 modulates immune cell distribution and activity within tumors, impacting gene networks involved in tumor progression, immune evasion, cell proliferation, and migration, thereby playing a vital role in the TIME (37, 38). Furthermore, the association between high GREM1 expression and various immune subtypes underscores its crucial role in TIME regulation (39).

Chemokines regulate immune cell migration, influence tumor progression, and recruit diverse immune cells (40). Previous studies suggest that GREM1 modulates chemokine expression and activity, shaping immune cell recruitment and function (41). Our research demonstrates an association between GREM1 expression and multiple chemokines, including CCL8, CCL21, CXCL10, CXCL11, and CXCL12. We speculate that GREM1 may regulate immune cell recruitment and localization in the TME by enhancing specific chemokine expression or activity, thus contributing to both antitumor immunity and tumor immune evasion. Additionally, we found that GREM1 is linked to various immune cell markers, including inflammation-promoting genes, macrophages, T-cell co-inhibition genes, TILs, and Tregs, underscoring its potential in immunotherapy development (33, 34, 42).

GREM1 regulates cell invasion and metastasis, both crucial factors impacting prognosis. Previous studies indicate that GREM1 is closely associated with ECM and stromal cell function, especially in ECM organization and structural composition (28, 43). GREM1 facilitates ECM degradation and remodeling, disrupting normal tissue architecture to create conditions favorable for tumor cell migration and invasion, and also impacts immune cell function within the TME (44). Further research suggests that GREM1 is strongly linked to several signaling pathways that facilitate tumor cell invasion and metastasis. Notably, elevated GREM1 expression is associated with activation of the PI3K-Akt signaling pathway, a key pathway in cell proliferation, survival, migration, and invasion (45, 46). By promoting the activation of specific signaling pathways, GREM1 may enhance tumor cell invasion and metastasis. Moreover, GREM1 modulates the TGF-β signaling pathway, further increasing the metastatic potential of tumor cells (14, 37). Correlation analyses indicate that GREM1 is significantly positively correlated with genes associated with tumor invasion and metastasis, including TWIST1 and PDGFRA. Specifically, as a key EMT regulator, TWIST1 plays a crucial role in the detachment of tumor cells from the primary site and their migration to other tissues. GREM1 accelerates the EMT process by synergistically interacting with these genes, thereby enhancing the invasion and metastatic capabilities of tumor cells (18, 46, 47).

Through *in vitro* experiments, we thoroughly investigated the pathogenic mechanisms of GREM1 in LUAD, uncovering its critical role in cell proliferation, migration, and EMT. Western blot analysis first showed that GREM1 expression was significantly higher in cancerous tissues than in adjacent non-cancerous tissues and generally elevated in various LUAD cell lines relative to normal cells. By overexpressing and silencing GREM1, we further validated its function across various cell lines. The experimental results demonstrated that GREM1 overexpression markedly promoted EMT in A549 cells, as evidenced by the downregulation of the epithelial marker E-cadherin and the upregulation of mesenchymal markers N-cadherin and Vimentin. This phenomenon was closely associated with enhanced migration and invasiveness of LUAD cells, suggesting that GREM1 plays a crucial role in driving the transition of LUAD cells toward a more invasive mesenchymal phenotype (27, 32). Conversely, GREM1 knockdown in H1650 cells led to upregulation of E-cadherin and downregulation of N-cadherin and Vimentin, further supporting GREM1’s role as an EMT-promoting factor. Additionally, we confirmed GREM1’s role in cell proliferation. In A549 cells, GREM1 overexpression significantly promoted proliferation, while its knockdown in H1650 cells markedly inhibited it. These findings suggest that GREM1 enhances cellular invasiveness by promoting EMT and supports tumor growth by regulating proliferation. Wound healing and Transwell assays further confirmed GREM1’s positive regulatory role in cell migration and invasion. GREM1 overexpression significantly enhanced migration and invasion in A549 cells, while its knockdown in H1650 cells notably suppressed these abilities. In the Transwell assay, GREM1 expression levels strongly correlated with cell migration and invasion capacities, suggesting that GREM1 may promote tumor metastasis by enhancing cell motility. These *in vitro* findings highlight GREM1’s critical regulatory role in LUAD. GREM1 facilitates cell invasion and migration by regulating the EMT process and is closely associated with malignant tumor behaviors through enhanced cell proliferation.

Additionally, elevated GREM1 expression may be linked to genomic instability in tumor cells, potentially facilitating tumor invasion and metastasis. Research indicates that GREM1 is closely associated with copy number variations (CNVs), a key indicator of genomic instability (48). These findings suggest that GREM1 may increase the adaptability and metastatic potential of tumor cells through the regulation of genomic instability (49).

Pan-cancer studies have shown that GREM1 is consistently overexpressed across various cancers, including BRCA, HNSC, KIRC, KIRP, THCA, and UCEC. This broad overexpression suggests that GREM1 plays a critical biological role across multiple cancers and is strongly associated with their initiation, progression, invasion, and prognosis (13, 50, 51). Univariate Cox analysis across multiple cancer cohorts has shown that high GREM1 expression is significantly associated with poor prognosis, particularly in cancers like KIRC, KIRP, malignant MESO, PAAD, and STAD. This indicates that GREM1 functions not only as a biomarker for specific cancers but also as a pan-cancer biomarker with extensive prognostic value. High GREM1 expression frequently correlates with lower survival rates, indicating it may serve as a key driver gene or regulatory factor in these cancers. Due to its high expression across multiple cancers and strong association with poor prognosis (35, 52, 53), GREM1 shows promise as a broadly applicable therapeutic target. Currently, inhibitors targeting GREM1, such as Ginisortamab (UCB6114) and TST003, have shown preliminary progress in several experimental models. The development of these inhibitors primarily hinges on the critical role of GREM1 in tumor progression. Ginisortamab, by blocking the interaction between GREM1 and BMP, restores BMP signaling, potentially suppressing tumor stem cell proliferation and self-renewal while reducing the risk of chemoresistance and relapse (54). On the other hand, TST003 significantly decreases tumor cell proliferation and spheroid formation capacity by inhibiting the FGFR1/MAPK signaling axis, demonstrating potential antitumor activity in various refractory solid tumor models (15). These preclinical studies not only confirm the efficacy of GREM1 inhibitors but also reveal potential mechanisms through which they modulate the TME, influencing immune cell infiltration and function.

Despite the extraordinary importance of GREM1 as a potential therapeutic target, its clinical application faces several challenges: 1. Specificity Challenges: Given that GREM1 plays a crucial role in tissue repair and cell development under normal physiological conditions, systemic inhibition of GREM1 may lead to a series of unintended side effects. These side effects could include, but are not limited to, interference with normal tissue regeneration and repair mechanisms, thereby posing potential risks to overall health (15, 27). 2. Tumor Heterogeneity: Significant inter-patient variability in tumor molecular characteristics introduces substantial uncertainty regarding the efficacy of GREM1-targeted therapies. This heterogeneity may result in varying therapeutic responses among patients, with some individuals potentially experiencing suboptimal outcomes, thereby limiting the broad applicability of this treatment strategy (55, 56). 3. Risk of Drug Resistance: Long-term reliance on single-agent GREM1 inhibitors might prompt tumor cells to activate alternative signaling pathways to evade drug effects, ultimately leading to resistance. The emergence of such resistance mechanisms could not only diminish initial therapeutic efficacy but also facilitate disease progression, adding complexity and challenges to long-term patient management.

To optimize the application of GREM1 as a therapeutic target and address these challenges effectively, the following strategies are proposed: Firstly, efforts should focus on designing highly specific small-molecule inhibitors and employing RNA interference technologies to ensure that these approaches target only the overexpressed GREM1 in tumors, thereby minimizing potential effects on normal tissues (15). Secondly, the potential of combination therapies should be explored, such as combining GREM1 inhibitors with other therapeutic modalities, including immune checkpoint inhibitors (e.g., PD-1/PD-L1 antibodies) or chemotherapeutic agents, to achieve synergistic enhancement of therapeutic efficacy and effectively overcome potential resistance (15, 54). Finally, by deeply integrating genomic, transcriptomic, and immunomic data, biomarker screening strategies should be implemented to accurately identify patient subgroups most likely to benefit from GREM1-targeted therapies, thereby improving the precision and personalization of treatments and providing more tailored and effective therapeutic options for patients.

In addition to the findings discussed above, it is crucial to compare our results with previous studies on GREM1, particularly regarding its dual role in tumor progression and immune regulation. Previous research has demonstrated that GREM1 regulates tumor progression through the BMP and TGF-β signaling pathways. For instance, in breast cancer, GREM1 acts as a BMP antagonist to inhibit the BMP/SMAD signaling pathway, thereby promoting EMT and tumor invasion (57). Similarly, in bladder cancer, GREM1 has been shown to drive tumor progression by activating the PI3K/AKT signaling pathway (46, 58). While these studies highlight the role of GREM1 in tumor proliferation, migration, and invasion, they do not fully address its impact on the immune microenvironment. Since research on GREM1 in LUAD is relatively limited, our study is the first to elucidate the role of GREM1 in regulating the tumor immune microenvironment. Specifically, we observed a significant correlation between GREM1 expression and immune cell infiltration, including macrophages and T cells, as well as its association with immune evasion mechanisms. These findings fill a critical gap in the literature, as existing studies largely overlook the immunological significance of GREM1 in LUAD.

Despite the preliminary findings of our study regarding the function of GREM1 in LUAD cells-validated through *in vitro* experiments that demonstrate its effects on cell proliferation, migration, and invasion-we must acknowledge the limitations in both the depth and breadth of the current research. In particular, there remains a lack of detailed elucidation of the molecular mechanisms by which GREM1 regulates immune cell recruitment and EMT. Furthermore, our study primarily relies on *in vitro* data, lacking robust *in vivo* experimental evidence to confirm the precise role of GREM1 within the TME. To address these limitations, future studies should consider constructing animal models to more directly observe the impact of GREM1 on tumor growth, immune cell infiltration, and the EMT process. Additionally, while we have derived conclusions with potential clinical translational value through integrated analyses of multi-omics data from independent cohorts, the role of GREM1 in LUAD prognosis and immunotherapy requires further validation in prospective patient cohorts, particularly those with adequate follow-up data and undergoing immunotherapy. Our study has revealed the close association of GREM1 with the invasiveness and metastasis of LUAD cells, as well as its interplay with the immune microenvironment, highlighting the critical role of GREM1 in LUAD pathogenesis and prognosis assessment. However, to optimize therapeutic strategies for LUAD patients-such as exploring the potential of combining GREM1-specific inhibitors with immune checkpoint inhibitors (ICBs)-it is imperative to delve deeper into the molecular mechanisms underlying these observations in both *in vitro* and *in vivo* systems. This would provide a more solid theoretical foundation for future clinical applications.

In summary, mechanistic analysis shows that GREM1 plays a critical role in tumor invasion, metastasis, and immune evasion. It acts through multiple pathways, including influencing the TME, regulating key signaling pathways, synergizing with other invasion-related genes, and promoting genomic instability. Bioinformatics analysis and *in vitro* experiments validated GREM1’s role in promoting proliferation, migration, and EMT in LUAD cells. Additionally, the complex relationship between GREM1 and the TIME, as well as its influence on immunotherapy response, underscores its importance as a therapeutic target. GREM1 not only serves as a promising therapeutic target but also suggests new directions for cancer treatment strategies. Inhibiting GREM1 expression or function could become a crucial approach to halting LUAD progression.

## 5 Conclusion

This study highlights the significant role of GREM1 in LUAD as a prognostic marker and therapeutic target. Our analysis demonstrates that high GREM1 expression is associated with poorer prognosis and increased invasiveness in LUAD. Furthermore, its correlation with immunosuppressive cell infiltration and immune checkpoint markers suggests a role in modulating the tumor immune microenvironment. These findings indicate that GREM1 could be a biomarker for ICB therapy. Future studies should explore the potential of GREM1 inhibitors, particularly in combination with ICIs, to improve treatment outcomes in LUAD patients. We observed that GREM1 is not only linked to tumor cell proliferation, migration, and EMT but also closely associated with immune cell infiltration within the TME. The correlation between GREM1 and immune checkpoint markers suggests a potential role in influencing LUAD’s immunotherapy response. By integrating gene expression data with immune and pathway analyses, we propose that GREM1 serves as a critical mediator in LUAD progression; targeting it may lead to improved patient outcomes, especially in combination with immunotherapeutic approaches.

However, this study has several limitations. First, while our bioinformatics analyses provided robust insights, further validation in clinical settings with larger patient cohorts is necessary to confirm GREM1’s prognostic value and therapeutic potential. Further *in vivo* studies are needed to explore the molecular mechanisms by which GREM1 modulates immune responses within the TME and its precise role in immunotherapy response. Lastly, although *in vitro* assays demonstrated GREM1’s impact on cell behaviors, translating these findings into clinical applications will require more extensive preclinical trials.

## Data Availability

The original contributions presented in the study are included in the article/**Supplementary Material**. Further inquiries can be directed to the corresponding author.
